# Study of the Polysaccharide Production by the Microalga *Vischeria punctata* in Relation to Cultivation Conditions

**DOI:** 10.3390/life12101614

**Published:** 2022-10-15

**Authors:** Olga Babich, Ekaterina Budenkova, Egor Kashirskikh, Vyacheslav Dolganyuk, Svetlana Ivanova, Alexander Prosekov, Veronika Anokhova, Anna Andreeva, Stanislav Sukhikh

**Affiliations:** 1 Institute of Living Systems, Immanuel Kant Baltic Federal University, A. Nevskogo Street 14, 236016 Kaliningrad, Russia; 2 Department of Bionanotechnology, Kemerovo State University, Krasnaya Street 6, 650043 Kemerovo, Russia; 3 Natural Nutraceutical Biotesting Laboratory, Kemerovo State University, Krasnaya Street 6, 650043 Kemerovo, Russia; 4 Department of General Mathematics and Informatics, Kemerovo State University, Krasnaya Street 6, 650043 Kemerovo, Russia; 5 Laboratory of Biocatalysis, Kemerovo State University, Krasnaya Street 6, 650043 Kemerovo, Russia

**Keywords:** *Vischeria punctata*, microalgae, nutrient medium, endopolysaccharides, exopolysaccharides, uronic acids, extraction, neutral sugars

## Abstract

*Vischeria punctata* is a unicellular microalga that has industrial potential, as it can produce substances with beneficial properties. Among them, endopolysaccharides (accumulated in cells) and exopolysaccharides (released by cells into the culture medium) are of particular interest. This study aimed to investigate the effect of nutrient medium composition on the growth of *V. punctata* biomass and the synthesis of polysaccharides by microalgae. The effect of modifying a standard nutrient medium and varying cultivation parameters (temperature, time, and extractant type) on the yield of exopolysaccharides produced by the microalgae *V. punctate* was investigated. The methods of spectrophotometry, ultrasonic extraction, and alcohol precipitation were used in the study. It was found that after 61 days of cultivation, the concentration of polysaccharides in the culture medium was statistically significantly higher (*p* <0.05) when using a Prat nutrient medium (984.9 mg/g d.w.) than BBM 3N (63.0 mg/g d.w.). It was found that the increase in the *V. punctata* biomass when cultivated on different nutrient media did not differ significantly. The maximum biomass values on Prat and BBM 3N media were 1.101 mg/g d.w. and 1.120 mg/g d.w., respectively. Neutral sugars and uronic acids were found in the culture media. It follows on from the obtained data that the modified PratM medium was more efficient for extracting polysaccharides from *V. punctata*. The potential of microalgae as new sources of valuable chemicals (polysaccharides), which can be widely used in technologies for developing novel functional foods, biologically active food supplements, and pharmaceutical substances, was studied.

## 1. Introduction

Humans have long used microalgae as organisms capable of producing biologically active substances. Polysaccharides, which have biological activity, are one of these substances [1,2,3]. Exopolysaccharides (EPSs), which are released into the culture medium, and endopolysaccharides, also known as bound polysaccharides, which can either accumulate inside of cells or be connected to the cell wall of microalgae, are the two main types of polysaccharides found in microalgae.

Depending on the growing conditions of microalgae, the composition of polysaccharides and their amount may vary. Particular attention is paid to obtaining polysaccharides from highly purified microalgae that are free of impurities. Therefore, the optimization of the extraction and purification of polysaccharides is an urgent problem. The optimization of extraction methods has already been investigated for the polysaccharides of red algae [4] and green algae *Dictyosphaerium* sp. [5].

Despite the high potential of *Vischeria* sp., there are not enough studies on its cultivation compared to other microalgae representatives. The genus *Vischeria* was recently shown to be identical to the genus Eustigmatos and these genera are now merged. Eustigmatos is mentioned in papers [6,7,8]; however, the exopolysaccharides of the microalgae *V. punctate* have received little attention [6]. Soils, subaerial space (for example, tree bark), and fresh waters are the habitats of the members of the genus *Vischeria*.

*Vischeria punctata* is a unicellular microalga that has industrial potential, as it can produce substances with beneficial properties [7]. In recent years, *Eustigmatophyceae*, which include *V. punctate*, have attracted increasing attention because they are biotechnologically promising in terms of biomass growth, production of lipids, pigments, and other compounds [9]. The screening of the biologically active substances of *V. punctate* made it possible to establish high levels of β-carotene (up to 5.9% dry weight) and a biomass production rate of up to 9.8 g/L in bubble column photobioreactors. These microalgae produce 0.28 g/L/day of lipids, the algae can be a source of nutraceuticals, or they can be used for biodiesel production. Additionally, the majority of the fat components are polyunsaturated fatty acids, and some of these algae have been found to contain significant amounts of valuable eicosapentaenoic acid [10]. Under stress conditions, these microalgae are known to be a superproducer of eicosapentaenoic fatty acid [11,12]. Schnepf et al. [13] notes that all Eustigmatophyceae are characterized by the presence of so-called refractive granules, which are a storage substance of a β-1-3-linked polysaccharide. The genus *Vischeria* and other members of the *Eustigmatophyceae* class can produce biologically active substances such as violaxanthin, lutein, and astaxanthin [14]. These substances have anti-inflammatory, antioxidant, and anti-cancer properties [15]. However, there are very few studies on the influence of the composition of the culture medium on the yield of exopolysaccharides. Sinetova et al. [11] discovered that the carbohydrate content of *V. punctata* cells grown on BBM 3N (Bold’s basal media) can reach around 100 mg per g of dry biomass. It can be assumed that the microalga *V. punctata* is also capable of producing EPSs. Thus, this study aims to investigate the effect of nutrient medium composition on the increase in *V. punctata* biomass and the synthesis of polysaccharides by microalgae. The effect of modifying a standard nutrient medium and varying cultivation parameters (temperature, time, and extractant type) on the yield of exopolysaccharides produced by the microalgae *V. punctate* is investigated. A number of studies [1] confirm that a decrease in nutrient concentrations in the culture medium contributes to increased exopolysaccharide production by microalgae. Since *V. punctata* is a photosynthetic microorganism, it is possible to conduct an experiment with extremely low nutrient concentrations.

EPSs, depending on the species, are formed simultaneously with the growth of microalgae. EPS production is affected by special conditions that may be associated with nutrient depletion, high salinity, high light levels, etc. [16]. Depending on the photosynthetic microorganisms, EPSs can be completely released into the medium or remain more or less tightly bound to the cells. EPSs are produced first as bound polymers and then gradually released as a result of some operating parameters. This assumption correlates with the fact that the exopolysaccharide/endopolysaccharide ratio increases with the age of the microalgae culture.

The unique rheological properties of microalgal EPSs allow them to be considered as new gel formers and thickeners. Exopolysaccharides from microalgae may find new uses because of their unique properties and projected future lower production costs. The biological activities of microalgal EPSs, such as their antibacterial, antioxidant, anti-inflammatory, antiparasitic, immunomodulatory, antitumor, and anticoagulant properties, have also been the subject of numerous studies, both in vitro and in vivo. All of these attractive features (a wide range of microalgae and cyanobacteria, unique structures, physicochemical properties, and/or biological activity) make compounds such as EPSs appealing for use in a variety of industries [17].

EPSs are usually extracted from the culture medium by alcohol precipitation [14,15]. The yield of EPSs varies depending on the length of the alcohol radical and the temperature of precipitation. Therefore, we chose alcohols with different structures (ethanol, butanol, and isopropanol) and different EPS extraction temperatures (ranging from −30 °C to +30 °C) [18]. Exopolysaccharide thermal stability is a key feature that opens up opportunities for the use of microalgal exopolysaccharides in the food, pharmaceutical, and cosmetic industries [18]. The degree of extraction and the yield of exopolysaccharides depend on the length of the alcohol radical. Exopolysaccharides obtained using alcohols with different radical lengths differ in water solubility and water-holding capacity, which provides stable characteristics for their use as hydrocolloids and stabilizers [6,10,19].

## 2. Materials and Methods

### 2.1. Microalgae Cultivation

*Vischeria punctata* H-242 strain was purchased from the collection of microalgae and cyanobacteria of the K.A. Timiryazev Institute of Plant Physiology of the Russian Academy of Sciences (IPPAS IPP RAS). By batch culture, the strain was grown in 250 mL conical flasks at 7500 ± 50 lux illumination (15 h light/9 h dark). Lighting was provided by an LED ProLine 580 LED lamp (Spectrum Brands, Inc., Melle, Germany) with white and red LEDs (8:1). Two nutrient media were used for cultivation (Table 1). BBM 3N medium was prepared according to Watanabe [20], and Prat medium was prepared according to Prat’s prescription [21], but with one modification (instead of FeCl_3_∙3H_2_O with a concentration of 0.001 g L^−1^, FeSO_4_∙7H_2_O with a concentration of 0.005 g L^−1^ was used in Prat medium). A modified Prat medium was also used, which contains a hundred times less KNO_3_, ten times less K_2_HPO_4,_ and MgSO_4_∙7H_2_O. Microalgae at an initial cell concentration of 0.1 g of raw biomass per 1 L of medium were cultivated in 100 mL of nutrient media. Cultures were aerated passively by gentle stirring on a PE-6110 magnetic stirrer (Oldis, Moscow, Russia). The cultures were incubated stationary for 61 days at room temperature (22–24 °C). All experiments were conducted with three repetitions.

### 2.2. Assessment of Cell and Biomass Growth

The concentration of cells in the culture medium was determined on a KFK-3KM spectrophotometer (Promyshlennyye ekologicheskiye laboratorii, St. Petersburg, Russia). The calibration curve of the dependence of the amount of biomass and the degree of absorption at 750 nm was obtained by sequential dilution of a concentrated suspension of the *V. punctata* culture [16]. Samples were taken twice a week to assess biomass growth, and the absorption level was measured at 750 nm with a KFK-3KM spectrophotometer (Promyshlennyye ekologicheskiye laboratorii, St. Petersburg, Russia). The biomass concentration (mg/g d.w.) of microalgae samples was determined using the calibration curve linear equation and used to plot the growth curve of microalgae grown on two different nutrient media. The transition of the culture into the exponential phase (log) of growth, as well as the completion of this phase, was determined by taking the logarithm of all absorption values and monitoring them depending on the time of cultivation. The specific growth rate (µ) of the culture, the number of cell divisions per day, and the biomass doubling time (T_d_) of microalgae were calculated using the formulas proposed by Griffiths et al. [22].

Specific culture growth rate (µ):

$$\mu =\frac{m_{1}-m_{0}}{t_{1}-t_{0}}$$

where m_1_—mass of microalgae culture at a certain point of growth (mg/g d.w.); *m_0_*—initial mass (mg/g d.w.); *t_1_*—time of sampling at a certain point in time (day); and *t_0_*—the start of culture growth.

Biomass doubling time (T_d_):

$$T_{d}=\left(t_{2}-t_{1}\right)\times \frac{\log\left(2\right)}{\log\left(\frac{q_{2}}{q_{1}}\right)}$$

where *t_1_*—start time of cultivation; *t_2_*—sampling time; *q_2_*—mass of microalgae culture at a certain point of growth (mg/g d.w.); and *q_1_*—initial mass (mg/g d.w.).

The linear equation of the calibration curve was used to calculate the biomass concentration (mg/g d.w.) of microalgae samples and to plot the growth curve of microalgae grown on two different nutrient media.

Calibration curve for dry weight. A culture of microalgae in the stationary phase of growth was taken. Sequential dilution with nutrient medium was performed. Each dilution was sampled with a volume of 100 mL. Samples were washed twice with distilled water. After the final centrifugation (3900 rpm for 10 min), the samples in the pre-weighed (to the fourth decimal place) dry container were left in an oven at 50 °C until they dried to constant weight (approximately overnight).

### 2.3. Quantification of Polysaccharides

The presence and quantitative assessment of polysaccharides from the microalga *V. punctata* were carried out by the anthrone–sulfate method at each stage of parameter selection. A total of 150 µL of an anthrone agent (a 0.1% solution of recrystallized anthrone in concentrated sulfuric acid) were added to each well of a microplate (DV-ekspert, Moscow, Russia) containing 50 µL of samples. Then the plates were placed in a Pozis RK-102 S refrigerator (Diamond Elektrik, Moscow, Russia) for 10 min at 4 °C. After cooling, the samples were incubated in an A-24 thermostat (Millab, Moscow, Russia) for 20 min at 70 °C. After heating, the samples were cooled to room temperature. Optical density was measured at 620 nm. A standard curve was plotted using sucrose solutions [23].

### 2.4. Alcohol Precipitation of Exopolysaccharides

Alcohol precipitation was used because when miscible alcohols are added in a solution of polysaccharides they can disturb the balance of interactions between polysaccharide–polysaccharide and polysaccharide–water molecules. This can cause polysaccharide aggregation and eventually precipitation [24]. Butanol was used as a water-insoluble alcohol for two-phase extraction of exopolysaccharides as an experiment.

The culture medium with microalgae cells was centrifuged at 3900 rpm for 20 min in a 1701 Hettich ROTINA 380 centrifuge (DV-ekspert, Moscow, Russia), and then the supernatant was collected and filtered through a paper filter with a pore size of 2–3 μm (Millab, Moscow, Russia). The filtrate was mixed with various alcohols (ethanol, butanol, and isopropanol) in various ratios (1:1, 1:2, and 1:3) and left to settle for 12 h at various temperatures (−30 °C … +30 °C in increments of 10 °C). After precipitation, the solutions were centrifuged at 3900 rpm in a centrifuge (DV-ekspert, Moscow, Russia); the supernatant was decanted, and the precipitate was dried in an Inei-6 lyophilic dryer (Institute of Biological Instrumentation, Russian Academy of Sciences, Pushchino, Russia) for 12 h at −20 °C, at a pressure of 0.350 mbar. After drying, the yield of isolated polysaccharides was determined gravimetrically. The isolated polysaccharide mass was then recalculated for dry biomass basis (mg/g d.w.) using the formula.

(1) $$m=\frac{m_{\mathrm{od}480}}{m_{d.w.}}$$

### 2.5. Ultrasonic Extraction of Bound Polysaccharides

The ultrasonic dispersion method was used to destroy the cell walls. The sediment of the culture liquid was dissolved in distilled water and the parameters of ultrasonic dispersion were selected in the UP200St ultrasonic unit (AntIs, St. Petersburg, Russia) with different power (20 W, 40 W, 60 W) and processing time (0.5, 1, 2, 3, 4 min) [25]. The polysaccharide mass was then recalculated using the anthrone–sulfate method for dry biomass (mg/g d.w.) using the formula (1).

### 2.6. Extraction of Bound Polysaccharides by Heat Treatment with the NaOH Addition

The method of increasing the pH and heat treatment was used to destroy the cell wall and release the endopolysaccharides. The sediment of the culture liquid was dissolved in distilled water and the parameters of heat treatment with the NaOH addition were selected. A 10% NaOH solution was added to change the pH of the medium, namely, the initial pH of the cell suspension, equal to 8, was adjusted to pH = 9, 10, 11. Then, samples of microalgae cell suspensions with different pH values were placed in an A-24 thermostat (Millab, Moscow, Russia) at different temperatures (25 °C, 45 °C, 65 °C, 85 °C, 100 °C) and kept for various time (60, 120, 180, 240 min). After each hour, a sample was taken, centrifuged, and the concentration of endopolysaccharides in the supernatant was determined by the anthrone–sulfate method. The polysaccharide mass was then recalculated using the anthrone–sulfate method for dry biomass (mg/g d.w.) using the formula (1)

### 2.7. Quantification of Uronic Acids

The level of uronic acids was determined by the carbazole method. To reduce the effect of neutral sugars on the result, 10 µL of a solution of sulfanilic acid in sulfuric acid (1%) was added to 250 µL of a microalgae suspension sample. Then, the test tubes were placed in a PE-4300 ice bath (PiterLab Company, St. Petersburg, Russia) and 1.5 mL of a solution of sodium tetraborate in sulfuric acid (0.1%) was added dropwise along the wall. After adding the solutions, the samples were placed in a PE-4300 boiling water bath (PiterLab Company, St. Petersburg, Russia) and heated for 6 min. Next, 50 μL of a carbazole solution in alcohol (0.1%) was added to the microalgae samples and again sent to a PE-4300 water bath (PiterLab Company, St. Petersburg, Russia) for 10 min. The tubes were cooled to room temperature and the optical density of the solutions was measured at 525 nm. The concentration of uronic acids was determined using a calibration curve built using galacturonic acid [26].

### 2.8. Quantification of Neutral Sugars

Neutral sugars were determined by the resorcinol–sulfate method. A total of 200 μL of a resorcinol solution recrystallized in butanol (6 mg/L) and 1 mL of 75% sulfuric acid were added to 200 μL of microalgae samples. The tubes were vortexed on a vortexer (DV-ekspert, Moscow, Russia) and heated in a PE-4300 water bath (PiterLab Company, St. Petersburg, Russia) at 90 °C for 30 min; then, the samples were cooled on the ice bath PE-4300 (PeterLab Company, St. Petersburg, Russia) in the dark for 30 min. Optical density was measured at 480 nm. The concentration of neutral sugars was determined according to a calibration curve plotted using glucose solutions [27].

The study used reagents and chemicals of an analytical or higher grade, which were purchased from Diaem, Moscow, Russia.

### 2.9. Statistical Analysis

All experiments and measurements were conducted with three repetitions. The results of the study are presented as a combination of mean and standard deviation (M ± SD). To test for a statistically significant intergroup difference, ANOVA analysis of variance was performed, followed by parametric Student’s and Tukey’s tests. The presence of a statistically significant difference was accepted under the condition of *p* <0.05. Statistical analysis was carried out using IBM SPSS Statistics 28.0.1 (IBM Corporation, New York, NY, USA), and the graphs were plotted in Excel (Microsoft Office, Microsoft Corporation, 15.0, 2016, Redmond, Washington, DC, USA).

## 3. Results

**Figure 1:**
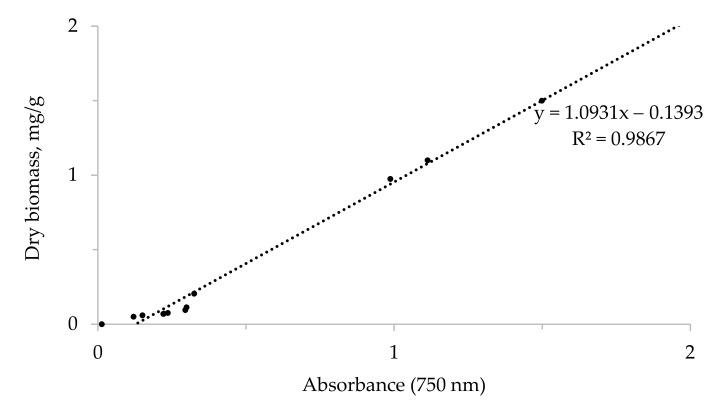
Calibration curve of adsorption at 750 nm versus *V. punctata* biomass (mg/g).

Figure 1 displays the calibration curve derived from the growth rates of microalgae biomass samples (excluding the nutrient medium).

**Figure 2:**
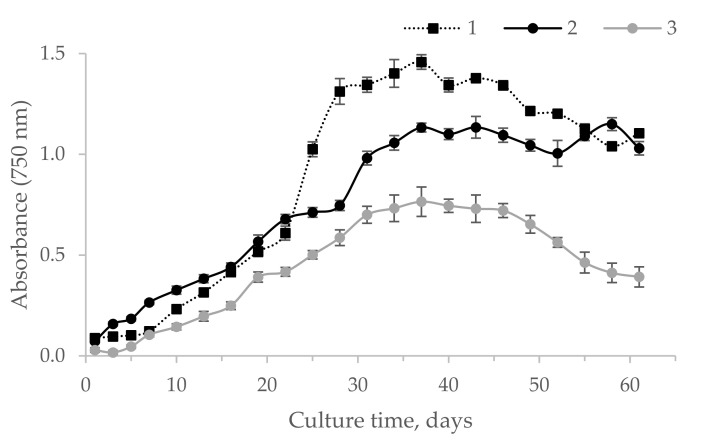
Growth curve of *V. punctata* on different nutrient media (n = 3): 1—BBM 3N; 2—Prat; 3—PratM.

**Figure 3:**
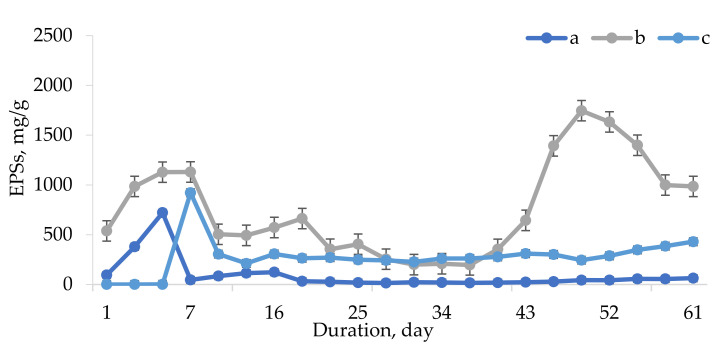
Curve of EPS production (mg/g d.w.) by microalga *V. punctata* during cultivation on different nutrient media (n = 3): a—BBM 3N; b—Prat; c—PratM.

Figure 2 shows the growth curves of *V. punctata* on three nutrient media. A culture in the initial stage of the stationary phase of growth was used to study this indicator. The analysis of Figure 3 shows that the greatest growth in the biomass of microalgae *V. punctate* was observed on the 20–40th days of cultivation.

According to numerous published data, it is known that under the influence of stress factors, microalgae can synthesis exopolysaccharides more intensively [28]. One of such factors is the lack of nutrients, primarily, sources of phosphorus and nitrogen, in the culture medium. Prat nutrient medium contains only four components at a low concentration, which, on the one hand, can contribute to a greater synthesis of exopolysaccharides by microalgae, and, on the other hand, makes this nutrient medium economically advantageous compared, for example, with the BBM 3N medium. Considering all of the above, as a third variant of the nutrient medium, a modified Prat medium (PratM), was used a hundred times less concentrated. We assume that the modified Prat medium will be the most effective for the production of polysaccharides during the cultivation of H-242. During the continuous cultivation of *V. punctata* in three different media (BBM 3N, Prat, and PratM), the number of cells first gradually increased and then decreased (Figure 2). The periodic determination of cell concentration in the medium showed that there were no statistically significant differences between the two media (*p* >0.05, ANOVA). The maximum cell concentration in the medium was 1.117 mg/g d.w. for the Prat medium (day 37) and 1.454 mg/g d.w. for the BBM 3N medium (day 28). From Figure 2, it can be noted that equal concentrations of microalgae biomass cultivated on two different media fell on the time interval between 30 and 35 days.

The exponential (logarithmic) phase of culture growth began on the 13th day and ended on the 34th with the Prat medium (biomass doubling time (µ) 0.043 day^−1^), and on the 28th day with BBM 3N (biomass doubling time (µ) 0.059 day^−1^). The number of divisions per day and the doubling time of the number of cells (Td) were 0.112 and 7.759, respectively, in the Prat medium, and 0.096 and 6.677 in the BBM 3N medium. A sharp decline in cell concentration was noted on the 37th day of cultivation on the Prat medium and on the 35th day of cultivation on the BBM 3N medium. After the end of the decline by day 43, the concentration of cells in the samples with both nutrient media remained at a relatively unchanged level. A statistically significant difference (*p* < 0.05, ANOVA) was found between the samples with two culture media in terms of the content of EPSs in them.

Figure 3 presents the curve of exopolysaccharide (EPS) production by the microalga *V. punctata* when grown on three different nutrient media. The measurement was carried out by the anthrone–sulfate method every three days throughout the entire cultivation period. The obtained data were corrected to the average value of dry weight during the measurement period. The analysis of Figure 3 leads us to the conclusion that *V. punctate* produced the most EPSs on the Prat medium on days 40–60 of cultivation. At the start of the experiment, there was a noticeable rise in exopolysaccharides per gram of dry biomass, which may be related to the activation of the adaptive potential of microalgae cells.

The content of EPSs in the intercellular space on the Prat medium (Figure 3) was higher (a maximum value of 1746 mg/g d.w.) compared to the BBM 3N medium (a maximum value of 720 mg/g d.w.). Regarding the Prat medium, a sharp increase in the EPS concentration was observed between the 40th and 50th days of cultivation, followed by a gradual decrease in concentration until the end of cultivation (day 61). In the case of BBM 3N, there was a slight slow increase in the concentration of EPSs in the medium throughout the entire cultivation time.

Table 2 shows the quantitative yields of the alcohol precipitation of exopolysaccharides by *V. punctate* in Prat (the 49th day of culture growth) with different alcohols, in different ratios, and at different temperatures.

Analyzing the data obtained (Table 2), we can conclude that the maximum yield of exopolysaccharides from *V. punctata* into the culture liquid was observed during precipitation with isopropyl alcohol in a ratio of 1:2 and 1:3 at a temperature of −20 °C, as well as in a ratio of 1:2 at 20 °C. The precipitation with butyl alcohol in a ratio of 1:2 at −20 °C also showed a high yield.

For the extraction of bound polysaccharides (BPSs) of *V. punctate* in Prat, two methods were chosen: ultrasonic dispersion and the thermal treatment of cells with an increase in pH. The parameters for ultrasonic dispersion were power and processing time. It was important to ensure that the cell solution did not overheat, as this could have affected the structure of the polysaccharides.

**Figure 4:**
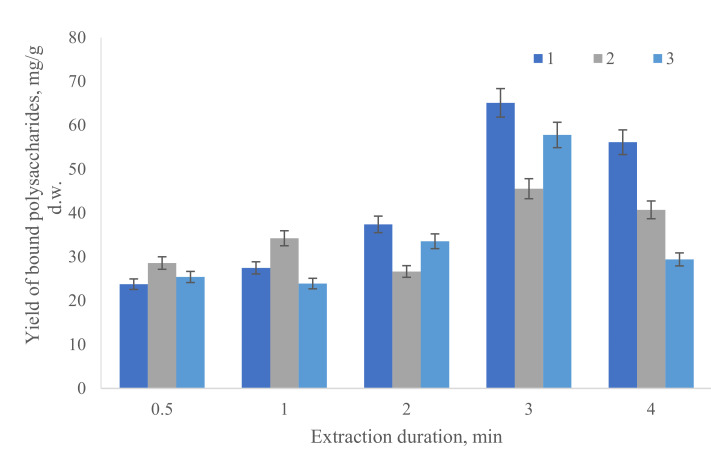
Yield dynamics of bound polysaccharides depending on the sonication power: 1–20 w; 2–40 w; 3–60 w. All values in a figure do not differ significantly from the rest (*p* > 0.05). Average values are presented (n = 3).

Figure 4 depicts the relationship between the yield of BPSs by *V. punctata* in Prat and the time and power of sonication.

At the beginning of the experiment, the total content of polysaccharides in the solution (20.2 mg/g d.w.) was determined by the anthrone–sulfate method. After the specified time, the sample was centrifuged and the total content of polysaccharides in the supernatant was determined (Figure 4). According to the results of the experiment, it became clear that the maximum yield (65.1 mg/g d.w.) of bound polysaccharides was observed when microalgae samples were treated with lower ultrasonic power (20 W) for 3 min (Figure 4). With a longer treatment time, the amount of bound polysaccharides decreased; this was probably due to the destruction of their structure under the influence of ultrasound.

Another method for extracting BPS is a chemical method with heat treatment. Sodium hydroxide was chosen as a chemical substance, which increases the pH of the medium. Initially, the pH of the medium was 8.3, it was then adjusted to 9, 10, and 11 to check the yield of bound polysaccharides at elevated pH. The temperature of heat treatment of microalgae solutions also varied. Endopolysaccharides by *V. punctata*, in Prat yield data, were checked by the anthrone–sulfate method every hour for four hours. Table 3 provides information on the polysaccharide amounts.

At pH = 11 for 60 min and 180 min of extraction, and at pH = 9, temperatures of 65 °C and 100 °C, with an extraction time of 180 min, the highest yields (132.2 mg/g d.w.) of endopolysaccharides from cells were observed (Table 3).

After selecting the parameters for the isolation of endopolysaccharides and exopolysaccharides, the methods for isolating endopolysaccharides were combined for a greater yield, and for the isolation of exopolysaccharides, a method was chosen with the maximum yield when selecting the parameters.

To compare the exopolysaccharides and endopolysaccharides of *V. punctata*, these microalgae were grown in three different media for 2 months. Then, *V. punctata* cells grown in different media were centrifuged to separate the culture fluid and cells.

**Figure 5:**
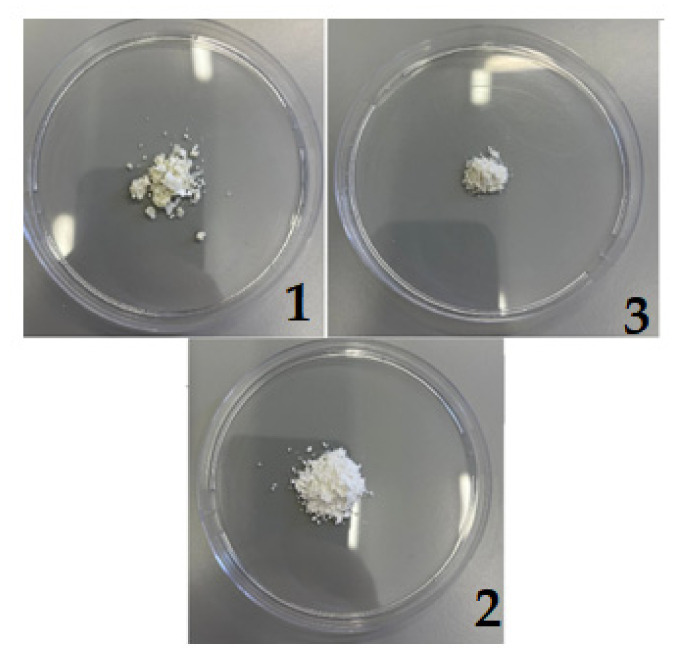
Appearance of exopolysaccharides isolated from *V. punctata* cultural liquid grown on different nutrient media: 1—BBM; 2—Prat; 3—PratM.

The total yield of *V. punctate* endopolysaccharides was 134.0 mg/g d.w. in the Prat medium, 93.0 mg/g d.w. in the PratM medium, and 102.0 mg/g d.w. in BBM 3N, which were determined gravimetrically. The total exopolysaccharide yield was 1611.9 mg/g d.w in the Prat medium, 240.0 mg/g d.w. in the PratM medium, and 40.0 mg/g d.w. in BBM 3N, which were determined gravimetrically. Figure 5 demonstrates dried exopolysaccharides from microalgae *V. punctate* grown in different media.

After the extraction of endopolysaccharides and exopolysaccharides, we performed a qualitative analysis for neutral sugars and uronic acids. Neutral sugars were determined by the resorcinol–sulfate method; uronic acids were determined by the carbazole method. The Table 4 presents the results in mg/g PS dry matter of the isolated polysaccharides.

The concentration of neutral sugars in endopolysaccharides was 440.6 g/mg SPS for the BBM 3N medium, 104.0 mg/g SPS for the Prat medium, and 272.3 mg/g SPS for the PratM medium. The concentration of uronic acids in endopolysaccharides was 95.0 mg/g SPS for the BBM 3N medium, 103.7 mg/g SPS for the Prat medium, and 95.4 mg/g SPS for the PratM medium.

The amount of neutral sugars in exopolysaccharides was 247.3 mg/g EPSs for the BBM 3N medium, 500.0 mg/g EPSs for the Prat medium, and 802.0 mg/g EPSs for the PratM medium. The concentration of uronic acids in exopolysaccharides was 96.0 mg/g EPSs for the BBM 3N medium, 96.8 mg/g EPSs for the Prat medium, and 92.4 mg/g EPSs for the PratM medium.

## 4. Discussion

This study looked at the ability of H-242 to produce EPSs when grown on two different nutrient media: BBM 3N and Prat media. Both media are recommended for the cultivation of H-242 (http://cellreg.org/Catalog_2020/Catalog%20NEW/IPPAS%20H-242.html (accessed on 30 August 2022)). The data obtained in the present study are consistent with those published by leading researchers [15,16,18,19,20,21,22].

One of the stress factors affecting the synthesis of exopolysaccharides by microalgae is the lack of phosphorus and nitrogen in the culture medium. A Prat medium is a nutrient-deficient medium. It contains only four components in low concentrations. This can increase the yield of exopolysaccharides and reduce the cost of this process.

Taking this fact into consideration, this study used a modified Prat medium (PratM medium), which had been diluted 100 times. It was assumed that *V. punctata* would synthesize exopolysaccharides even more efficiently on the PratM medium than on the standard Prat and BBM 3N media. From Figure 2, it can be noted that the equal concentrations of biomass of microalgae cultivated on two different media fell on the time interval between 30 and 35 days. The maximum cell concentration in the medium was 1.101 g L^−1^ for the Prat medium (day 37) and 1.120 g/g d.w. for the BBM 3N medium (day 28). During the continuous cultivation of *V. punctata* on three different media (BVM 3N, Prat, and PratM), the number of cells first gradually increased and then decreased (Figure 2). The periodic determination of cell concentration in the medium showed no statistically significant differences between the two media (*p* > 0.05, ANOVA).

The culture growth phase, as can be seen from Figure 2, began on the 13th day and ended on the 34th day on the Prat medium (biomass doubling time (dt) 0.043 day^−1^), and on the 28th day on the BBM 3N medium (biomass doubling time (dt) 0.059 day^−1^). The number of divisions per day and the doubling time of the number of cells (Td) were 0.112 and 7.759, respectively, in the Prat medium and 0.096 and 6.677 in the BBM 3N medium. A sharp decrease in cell concentration was noted on the 37th day of cultivation on the Prat medium and on the 35th day of cultivation on the BBM 3N medium. After the end of the decline by the 43rd day, the concentration of cells in the samples with both nutrient media remained at a relatively unchanged level. A statistically significant difference (*p* < 0.05, ANOVA) was revealed between the samples with two nutrient media in terms of the content of EPSs in them.

The cultivation of microalgae samples showed the presence of a lag phase, an exponential (logarithmic) phase, a linear growth phase, a growth deceleration phase, a stationary phase, and a dying phase. Growth dynamics depended on the amount of dilution and the nutrient medium used. Under certain conditions (the constancy of the biochemical composition, the age structure of the cell population, the concentration of metabolites, etc.), the growth dynamics after dilution repeated the previous dependence. Thus, it is possible to control the processes of microalgae growth by selecting cultivation parameters. Furthermore, only culture density and time can be used to control growth, as determined by the main kinetic characteristics of growth (growth rate, specific growth rate, specific respiration rate, efficiency of photobiosynthesis, etc.) [18].

In the course of the experiment, we found that the maximum concentration of exopolysaccharides was achieved during cultivation on the Prat nutrient medium at the initial (days 3–10) lag stage and at days 43–50 of cultivation.

After the cultivation of the microalga *V. punctata*, we separated the cells from the supernatant by centrifugation and selected the parameters for the extraction of endopolysaccharides and exopolysaccharides.

The data presented in Table 2 indicate that the use of ethanol as an extractant resulted in the lowest exopolysaccharide yield. When using isopropanol and butanol, the yield of exopolysaccharides was maximum at an extraction module of 1:2. The yield of exopolysaccharides was influenced by high and low temperatures. The highest yield of exopolysaccharides was observed at +20 °C and −20 °C.

Another important metabolite of microalgae was endopolysaccharides, which are produced inside the cells. For example, *Porphyridium cruentum* endopolysaccharides have an antioxidant effect [19], and samples of *Arthronema africanum* and *Nostoc commune* endopolysaccharides have antimicrobial and antioxidant effects [20]. Bound polysaccharides, according to the experiments described earlier, were extracted from cells by physical methods, for example, sonication [21], as well as various chemical agents [16,22].

For the extraction of bound polysaccharides (BPS) of *V. punctata*, two methods were chosen: ultrasonic dispersion and thermal treatment of cells with an increase in pH. The parameters for ultrasonic dispersion were power and processing time. It was important to ensure that the cell solution did not overheat, as this could have affected the structure of the polysaccharides. At the beginning of the experiment, the total content of polysaccharides in the solution (20.1 mg/g d.w.) was determined by the anthrone–sulfate method. After the specified time, the sample was centrifuged and the total content of polysaccharides in the supernatant was determined (Figure 4). According to the results of the experiment, it became clear that the maximum yield (65.1 mg/g d.w.) of bound polysaccharides was observed when microalgae samples were treated with lower ultrasonic power (20 W) for 3 min (Figure 4). With a longer treatment time, the amount of bound polysaccharides decreased, probably due to the destruction of their structure under the influence of ultrasound.

Another method for extracting BPS is a chemical method with heat treatment. Sodium hydroxide was chosen as a chemical substance that increases the pH of the medium. Initially the pH of the medium was 8.3, it was adjusted to 9, 10, and 11 to check the yield of bound polysaccharides at elevated pH. The temperature of heat treatment of microalgae solutions also varied (Table 3). At pH = 11 for 60 min and 180 min of extraction and at pH = 9, temperatures of 65 °C and 100 °C, with an extraction time of 180 min, the highest yields of endopolysaccharides from cells were observed. After the experiments, the methods for isolating endopolysaccharides were combined for a greater yield, and, for the isolation of exopolysaccharides, a method was chosen with the maximum yield when selecting rational parameters. The cells of the microalga *V. punctata* grown on the studied media were separated from the culture liquid by centrifugation. The culture fluid was used to isolate exopolysaccharides, and the cells themselves were used to extract endopolysaccharides. In a study by Costa et al. [29], the modes of the extraction of various microalgae are discussed. Polysaccharides were extracted under the following conditions: *Spirulina platensis*—ultrasonic treatment at pH 7–11 [30]; *Chlorella pyrenoidosa*—ultrasonic extraction [31]; *Chlorella vulgaris*—hydration in a sodium acetate buffer with cysteine and ethylenediaminetetraacetic acid (EDTA) (pH 5.0) and incubation with raw papain at 60 °C [32]; *Phaeodactylum tricornutum, Porphyridium* sp., *Dunaliella salina,* and *Arthrospira platensis*—the extraction of dry biomass under the influence of microwave radiation at 90 °C [33]; *Neochloris oleoabundans*—the heating (80 °C) and concentration of a cell-free medium at a reduced pressure and temperature at 60 °C [34]; and *Haematococcus pluvialis*—ultrasonic extraction. When using these types of extraction, the yield of polysaccharides ranged from 2.5% to 16.7% [21,23,24,25,26,27]. The biomass content in our study ranged from 0.045 ± 0.001 mg/g d.w. on the BBM 3N medium up to 1.17 ± 0.023 mg/g d.w. on the Prat medium. In studies [29,31,33,34], the biomass yield ranged from 0.03 mg/g d.w. up to 1.20 mg/g d.w. on various media.

After selecting the extraction parameters for endopolysaccharides and exopolysaccharides, we compared the qualitative composition of endopolysaccharides and exopolysaccharides in the cells of the *V. punctate* microalgae cultured in different media. The obtained polysaccharides were extracted according to the best parameters, which are described above.

For the qualitative determination of the composition of the obtained polysaccharides, the carbazole method was used to determine uronic acids and the resocine–sulfate method was used to determine neutral sugars. According to the data obtained, it can be concluded that the composition of polysaccharides varied depending on the growing medium of the microalgae *V. punctate*. Endopolysaccharides contained fewer neutral sugars in the widely used Prat medium than in the PratM and BBM 3N media. Perhaps it depends on the internal oxidative processes of cells. However, the content of uronic acids in these endopolysaccharides was higher when compared to the endopolysaccharides of cells in other media (Table 4). In microalgae, uronic acids are bound to the fibrous component of the plant cell. Uronic acids are found in non-cellular structural polysaccharides and derivatives, such as hemicellulose, pectins (land plants), and alginates (sea plants), as well as in non-structural polysaccharides, such as mucus, seed gums, plant secretions, and microbial gums (xanthan). These substances are also called soluble dietary fiber. The most common uronic acids in plants are d-galacturonic and d-glucuronic acids. Glucuronic acid is a structural unit of heparin, hyaluronic acid, etc. In the body, uronic acids perform an important function: they bind foreign and toxic substances, and, in the form of glucuronides, remove them from the body along with urine [28]. Uronic acids, especially D-galacturonic and D-glucuronic acids, are highly valuable chemicals used in the pharmaceutical, cosmetic, and food industries as antioxidants, as detoxifying and inactivating agents of various substances in the human body, ascorbic acid precursors, and chelating agents with anti-cancer properties. D-glucuronic acid is also used as a building block of hyaluronic acid and as a cosmetic ingredient in moisturizing and protective skincare cosmetic creams [35].

The composition of *V. punctata* EPSs also changed depending on the microalgae growing conditions. The highest content of neutral sugars in EPSs isolated into the PratM medium, with the lowest isolating into the BBM 3N (Table 4). An earlier increase in biomass when using the BBM 3N medium may be due to the greater nutritional value of the composition of this medium, compared to the Prat medium. The BBM 3N medium consisted of 13 components, while Prat medium consisted of only 4 (Table 1). At the same time, the concentration of the components of the Prat medium was lower (with the exception of FeSO_4_) by about 7 times than in the BBM 3N. In addition, the media differed in their source of nitrogen: KNO_3_ in the Prat medium and NaNO_3_ in BBM 3N. In the absence of nutritional deficiency (such as in the BBM 3N medium), cell metabolism was shifted towards the synthesis of carbohydrates, and the processes of photosynthesis and biomass growth were carried out more intensively than the synthesis and accumulation of substances such as lipids and carbohydrates.

The optimization of the nutrient medium plays a very important role in the economic feasibility of the bioprocess and in the isolation of exopolysaccharides [34]. The study [36] showed that the presence of pure nitrogen in nutrient media during the cultivation of *Cyanothice* sp. CCY 0110 has been shown to improve the production of EPSs. Although, the highest levels of EPSs from *Nostoc* sp. BTA97 and *Anabaena* sp. BTA990 were obtained in the presence of combined nitrogen sources [37]. The most common nitrogen source was NaNO_3_ [31,33,38], although KNO_3_ [34,39,40] also showed a positive correlation with EPS production. Interestingly, among the four different nitrogen sources, urea contributed the most to EPS production by *Nostoc flagelliforme* [41]. It was reported [28] that the absence of inorganic nitrogen, i.e., diazotrophic conditions, had a positive effect on the production of EPSs in some heterocyst-forming [42,43,44] and unicellular species [45].

The most commonly used inorganic carbon source is CO_2_ (0.05–10.00% by volume) in combination with air [28]. It was used to control the pH of the medium. It was found that NaHCO_3_ had a positive effect on the production of EPSs from *Arthropira platensis*, and, at the same time, it also contributed to an increase in the biomass yield of microalgae [46]. Some authors have evaluated the effect of using organic carbon sources. Many authors have reported enhanced EPS production under salt stress in the presence of NaCl [31,33,38]. It was shown that trace elements, such as MgSO_4_ [33], K_2_PHO_4_ [39,47], and CaCl_2_ [30], had a significant effect on the EPS yield, while no effect was found for MgCl_2_ and NaH_2_PO_4_ [45].

Various methods are currently used to isolate EPSs from microalgae biomass [16,17]. In the study [16], membrane filtration, dialysis, tangential ultrafiltration, or selective alcohol precipitation were used for these purposes. Alcohol precipitation is widely used in industry to purify the microalgal EPSs, and the possibility of alcohol recycling makes this process environmentally friendly. The main disadvantage of its use for the extraction of EPSs from microalgae is the low purity of the final product. The results of the study showed that tangential ultrafiltration with a molecular weight cutoff of 300 kDa using a polyethersulfone membrane was the most effective method. The same conclusion was made in the study [17]. These authors compared three concentration/precipitation methods (ethanol precipitation, Amicon cell diafiltration, and tangential ultrafiltration) for the isolation of EPSs produced by microalgae. The results showed that filtration methods are the most effective for isolating EPSs from the culture medium. However, it was noted that the use of membrane technologies is difficult due to the high viscosity of microalgae suspensions and the high cost of membranes [16].

## 5. Conclusions

The selection of the physicochemical parameters for the isolation of *V. punctata* endopolysaccharides and exopolysaccharides was performed in this study for the first time. A greater yield of EPSs was obtained by extraction with isopropyl alcohol in a ratio of 1:2 at room temperature, while BPSs were obtained by sonication at a power of 20 W for 3 min, at pH 9, and a temperature of 100 °C. The highest total yield of endopolysaccharides and exopolysaccharides was observed for *V. punctata* grown under the recommended rational conditions on the Prat medium and amounted to 134 mg/g d.w. SPS and 1611.9 mg/g d.w. EPSs, respectively. When using the BBM 3N medium and the modified PratM medium, a lower yield of exopolysaccharides was observed compared to the Prat medium. When cultivating on BBM 3N, the concentrations of endopolysaccharides and exopolysaccharides were 102 mg/g d.w SPS and 40 mg/g d.w. EPSs, and when cultivating on PratM, they were 93 mg/g d.w. SPS and 240.01 mg/g d.w. EPSs, respectively. A quantitative analysis was also performed for the content of neutral sugars and uronic acids. Based on the results, it can be concluded that the modified PratM medium is the most effective for extracting polysaccharides from *V. punctata* under optimized conditions.

Microalgae polysaccharides have promising biological properties for applications in various industries, especially in the food, pharmaceutical, and cosmetic industries [15]. Valuable chemicals (polysaccharides) of microalgae can be widely used in technologies for creating new types of functional food products, biologically active food supplements, and pharmaceutical substances. These products can be recommended for lowering the level of cholesterol in human blood and, thus, for preventing the development of atherosclerosis [4,12,28,29].

## Figures and Tables

**Table 1:** The composition of nutrient media for autotrophic cultivation of microalgae.

Component, g L^−1^	Medium		
	BBM 3N	Prat	PratM *
**NaNO_3_**	0.75	-	-
**KNO_3_**	-	0.1	0.001
**KH_2_PO_4_**	0.175	-	-
**K_2_HPO_4_**	0.075	0.01	0.001
**MgSO_4_∙7H_2_O**	0.075	0.01	0.001
**CaCl_2_∙2H_2_O**	0.025	-	-
**NaCl**	0.025	-	-
**FeSO_4_∙7H_2_O**	0.005	0.005	0.005
**H_3_BO_3_**	2.86	-	-
**MnCl_2_∙4H_2_O**	1.81	-	-
**ZnSO_4_∙7H_2_O**	0.222	-	-
**MoO_3_**	0.018	-	-
**NH_4_VO_3_**	0.023	-	-

* Modified Prat nutrient medium.

**Table 2:** Selection of parameters for the EPS extraction from the culture medium.

Solvent	Solvent Purity, %	Extraction Module, (Sample: Alcohol)	Precipitation Temperature, °C	Yield, mg/g d.w.
				H-242
Ethanol	96	1:1	20	104.5 ± 3.1a
			10	179.1 ± 5.3b
			0	179.1 ± 5.3b
			−10	253.7 ± 7.6c
			−20	179.1 ± 5.3a
Ethanol	96	1:2	20	806.0 ± 24.1a
			10	194.0 ± 5.8b
			0	253.7 ± 7.6c
			−10	626.9 ± 18.7d
			−20	507.5 ± 15.2e
Ethanol	96	1:3	20	925.4 ± 27.7a
			10	343.3 ± 10.9b
			0	432.8 ± 12.9c
			−10	44.8 ± 1.3d
			−20	74.6 ± 2.2e
Isopropanol	99	1:1	20	328.4 ± 9.8a
			10	358.2 ± 10.7a
			0	432.8 ± 12.9b
			−10	59.7 ± 1.8c
			−20	74.6 ± 2.2d
Isopropanol	99	1:2	30	1059.7 ± 31.7a
			20	1611.9 ± 48.3b
			10	507.5 ± 15.2c
			0	806.0 ± 24.2d
			−10	716.4 ± 21.4d
			−20	1597.0 ± 47.9b
			−30	716.4 ± 21.4d
Isopropanol	99	1:3	20	850.7 ± 25.5a
			10	955.2 ± 28.6a
			0	1134.3 ± 34.0b
			−10	641.8 ± 19.2c
			−20	1597.0 ± 47.9d
			−30	1194.0 ± 35.8b
Butanol	99	1:1	20	477.6 ± 14.3a
			10	955.2 ± 28.6b
			0	373.1 ± 11.2c
			−10	985.1 ± 29.5b
			−20	343.3 ± 10.3c
Butanol	99	1:2	20	820.9 ± 24.6a
			10	1597.0 ± 47.9b
			0	820.9 ± 24.6a
			−10	985.1 ± 29.5c
			−20	1611.9 ± 48.3b
			−30	1044.8 ± 31.3c
Butanol	99	1:3	30	820.9 ± 24.6a
			20	940.3 ± 28.2b
			10	179.1 ± 5.3c
			0	223.9 ± 6.7c
			−10	597.0 ± 17.9d
			−20	223.9 ± 6.7c

Values in a column followed by the same letter do not differ significantly from the rest (*p* > 0.05). Average values are presented (n = 3).

**Table 3:** The extraction parameters selection for bound polysaccharides, mg/g d.w.

pH	T, °C	Extraction time, min				
		25	45	65	85	100
9	60	66.8 ± 2.0a/a	57.9 ± 1.7a/a	81.7 ± 2.4a/b	78.7 ± 2.3a/b	74.3 ± 2.2a/b
	120	89.1 ± 2.6b/a	78.7 ± 2.3b/b	80.2 ± 2.4a/ab	86.2 ± 2.5a/a	86.2 ± 2.6b/a
	180	77.3 ± 2.3c/a	75.8 ± 2.2b/a	107.0 ± 3.2b/b	101.1 ± 3.1b/b	112.9 ± 3.4c/b
	240	63.9 ± 1.9a/a	50.5 ± 1.5a/b	54.9 ± 1.6c/b	56.5 ± 1.7c/b	63.9 ± 1.9a/a
10	60	41.6 ± 1.2a/a	54.9 ± 1.5a/b	47.5 ± 1.4a/a	69.8 ± 2.1a/c	83.2 ± 2.5a/d
	120	65.4 ± 1.9b/a	72.8 ± 2.1b/a	95.1 ± 2.8b/b	83.2 ± 2.5b/c	95.1 ± 2.8b/b
	180	71.3 ± 2.1b/a	69.8 ± 2.1bc/a	80.2 ± 2.4b/b	84.7 ± 2.5b/b	89.2 ± 2.6ab/b
	240	56.5 ± 1.7c/a	65.4 ± 2.0c/b	59.4 ± 1.8c/ab	74.3 ± 2.2a/c	60.9 ± 1.8c/ab
11	60	57.9 ± 1.7a/a	43.1 ± 1.3a/b	89.2 ± 2.6a/c	60.9 ± 1.8a/	112.9 ± 3.4a/
	120	87.7 ± 2.6b/ab	84.7 ± 2.5b/a	92.1 ± 2.7a/b	65.4 ± 1.9a/c	102.5 ± 3.0a/b
	180	72.8 ± 2.2c/a	65.4 ± 1.9c/b	77.3 ± 2.3b/a	86.2 ± 2.6b/c	132.2 ± 3.9b/d
	240	49.0 ± 1.4d/a	57.9 ± 1.7d/b	66.9 ± 2.0c/c	74.3 ± 2.2c/c	83.2 ± 2.4c/d

Values in a column/row the same lettery do not differ significantly from the rest (*p* > 0.05). The average values of the exopolysaccharide yields, mg/g d.w., are presented (n = 3).

**Table 4:** Relative content of polysaccharides in the studied samples.

Indicator	Endopolysaccharides			Exopolysaccharides		
	BBM 3N	Prat	PratM	BBM 3N	Prat	PratM
Neutral sugars, mg/g PS	440.6 ± 13.2a	104.0 ± 3.1b	272.3 ± 8.1c	247.5 ± 7.4a	500.0 ± 15.0b	802.0 ± 24.0c
Uronic acids, mg/g PS	95.0 ± 2.8a	103.7 ± 3.1a	95.4 ± 2.7a	96.0 ± 2.8a	96.8 ± 2.8a	92.4 ± 2.7a

Values in a row followed by the same letter do not differ significantly from the rest (*p* > 0.05). Average values are presented (n = 3).

## Data Availability

The raw data supporting the conclusions of this article will be made available to any qualified researcher on request.
